# Effects of selective serotonin reuptake inhibitors on glaucoma: A nationwide population-based study

**DOI:** 10.1371/journal.pone.0173005

**Published:** 2017-03-03

**Authors:** Vincent Chin-Hung Chen, Mei-Hing Ng, Wei-Che Chiu, Roger S. McIntyre, Yena Lee, Tsang-Yaw Lin, Jun-Cheng Weng, Pau-Chung Chen, Chung-Yao Hsu

**Affiliations:** 1 Department of Psychiatry, Chang Gung Medical Foundation, Chiayi Chang Gung Memorial Hospital, Chiayi, Taiwan; 2 Department of Psychiatry, School of Medicine, Chang Gung University, Taoyuan, Taiwan; 3 Tsaotun Psychiatric Center, Ministry of Health and Welfare, Nan-Tou County, Taiwan; 4 PhD Student of Institute of Medicine, Chung Shan Medical University, Taichung, Taiwan; 5 Department of Psychiatry, Cathay General Hospital, Taipei, Taiwan; 6 College of Medicine, Fu Jen Catholic University, Taipei, Taiwan; 7 Mood Disorders Psychopharmacology Unit, University Health Network, University of Toronto, Toronto, Canada; 8 Departments of Psychiatry and Pharmacology, University of Toronto, Toronto, Canada; 9 Department of Medical Imaging and Radiological Sciences, Chung Shan Medical University, Taichung, Taiwan; 10 Institute of Occupational Medicine and Industrial Hygiene, National Taiwan University College of Public Health, Taipei, Taiwan; 11 Department of Neurology, Kaoshiung Medical University Hospital, Kaoshiung, Taiwan; 12 Department of Environmental and Occupational Medicine, National Taiwan University Hospital, Taipei, Taiwan; Yeshiva University Albert Einstein College of Medicine, UNITED STATES

## Abstract

**Background:**

Selective serotonin reuptake inhibitors (SSRIs) are one of the most commonly prescribed classes of antidepressants. Glaucoma is the second leading cause of blindness globally and iatrogenic glaucoma has been implicated across disparate medication classes. Available studies that have sought to determine the association between SSRI exposure and glaucoma have provided mixed results. The aim of the study herein was to investigate whether an association exists between SSRI exposure and glaucoma incidence.

**Methods:**

Glaucoma cases were identified from Taiwan’s National Health Insurance Research Database with a new primary diagnosis of glaucoma between 1997 and 2009. The date wherein the cases were diagnosed with glaucoma was operationalized as the index date. The control group was comprised of individuals within the database who were not diagnosed with glaucoma. 15,865 glaucoma cases were compared to 77,014 sex-, age-, residence- and insurance premium-matched controls on measures of prescribed duration and dosage of SSRIs up to 365 days before index date to proxy SSRIs exposure.

**Results:**

Individuals receiving SSRIs were at greater risk of glaucoma incidence (OR = 1.39; 95% CI = 1.29–1.50); the foregoing increased likelihood was reduced after adjusting for confounding variables (aOR = 1.09; 95% CI = 1.00,1.18). SSRI treatment of longer duration (i.e. >365 days) and higher doses (≥1 defined daily dose) were associated with greater risk of glaucoma incidence (aOR = 1.36; 95% CI = 1.08–1.71). Subgroup analysis showed that the effect of SSRIs on glaucoma was limited to individuals younger than 65 years of age (aOR = 1.37; 95% CI = 1.25–1.50), without diabetes (aOR = 1.39; 95% CI = 1.27–1.52), without hypertension (aOR = 1.46; 95% CI = 1.31–1.63) or hypercholesterolemia (aOR = 1.35; 95% CI = 1.23–1.48).

**Conclusion:**

Treatment with SSRIs was associated with greater risk of having a diagnosis of glaucoma, particularly in individuals with longer duration and/or higher average dose of SSRI. Our findings suggest that individuals receiving SSRIs treatment for extended periods of time and/or at relatively higher therapeutic doses should be monitored for symptoms associated with glaucoma.

## Introduction

Major depressive disorder is one of the leading causes of global disability-adjusted life years (DALYs) [1], having increased from the 15th in 1990 to the 11th rank as a leading cause of DALYs globally in 2010 [2]. Selective serotonin reuptake inhibitors (SSRIs) are one of the most widely prescribed treatments for mood disorders and other conditions [3]. During the past two decades, antidepressant prescription, as well as antidepressant co-prescription, has increased and continues to increase amplifying the need for surveillance of possible safety concerns with increased exposure [4,5].

Glaucoma, a set of ocular disorders characterized by intraocular pressure-associated optic neuropathy, is the second leading cause of blindness globally after cataracts [6,7]. Agents from several different classes of antidepressants (e.g. amitriptyline, imipramine, mianserin hydrochloride, paroxetine, fluoxetine, fluvoxamine, citalopram, escitalopram) have been reported to be associated with increased intraocular pressure and risk for glaucoma [8,9,10]. For example, tricyclic antidepressants are known to have anticholinergic side effects and are frequently associated with glaucoma in predisposed individuals. Selective serotonin reuptake inhibitors (SSRIs), one of the most commonly prescribed medications globally, have also been reported to be associated with secondary glaucoma [11]. It has been hypothesized that SSRIs may increase intraocular pressure via serotonergic effects on ciliary body muscle activation and pupil dilation [3,12]. However, available studies that have sought to determine the association between SSRIs exposure and glaucoma have provided mixed results.

Between 1992 and 2001, the Australian Adverse Drug Reactions Advisory Committee (ADRAC) received 11 reports of elevated intraocular pressure following SSRIs treatment with onset occuring within 6 months of SSRIs treatment initiation [13]. In a recent study, patients prescribed SSRIs were noted to have a 5.8-fold elevation in risk for acute angle closure glaucoma within 7 days of SSRIs treatment initiation [14]. Interpretings the foregoing studies however, are limited by the relatively few studies reporting on long-term exposure to SSRIs and glaucoma risk. A separate study reported that long-term use (>365days) of SSRIs was not associated with elevated risk of primary open-angle glaucoma (POAG) or primary angle-closure glaucoma (PACG) in patients with depression [15].

The widespread prescription of antidepressants by multiple healthcare providers invites the need for multi-disciplinary awareness and up-to-date knowledge of any putative risk of glaucoma related to antidepressant exposure [16]. Herein, we primarily aim to investigate the possible association between glaucoma and SSRIs exposure (i.e. duration and dosing) using a case-control study design with data from Taiwan’s nationwide, population-based database. Our analysis was not confined to any particular diagnosis and instead looked exclusively at antidepressant prescription.

## Materials and methods

### Data source

The data for our analysis was retrieved from the Longitudinal Health Insurance Database (2005), which is derived from Taiwan’s Bureau of National Health Insurance records and maintained by the Taiwanese National Health Research Institute. With the introduction of Taiwan’s government launched the single-payer National Health Insurance (NHI) program in 1995. By December 2008, 22.918 million individuals nation-wide (i.e., 99.5% of the Taiwanese population) were enrolled into the NHI program [16]. Claims data collected as part of the NHI Registry for Beneficiaries (e.g. ambulatory care, hospital inpatient care and prescription claims data) comprise the National Health Insurance Research Database (NHIRD), which has been described and validated for patient data accuracy in previous publications [17,18].

In addition to patient care data, the NHIRD provides information on insurance premium categories, which can be used to proxy patient economic status. All individuals are classified into three ranked categories: fixed premium and dependents; income less than 20,000 New Taiwan Dollars (NTD) per month (1US $ = 32.1 NTD in 2008); and income NTD 20,000 or more per month. The ‘fixed premium and dependents’ group is comprised of individuals requiring social welfare support (e.g. low-income citizens, veterans) and their dependent(s) without regular income. Urbanization levels in Taiwan were divided into four strata according to Taiwan National Health Research Institute publications, from the “least urbanized” to the “most urbanized” communities. The database has been validated previously as part of pharmaco-epidemiological studies and published in peer-reviewed journals [19,20,21].

Our study utilized the Longitudinal Health Insurance Database 2005 (LHID2005), which includes all original claims data between 1997 and 2009 related to the care of 1,000,000 individuals (i.e. equal to approximately 5% of the total population of Taiwan). The foregoing set of individuals were randomly sampled from the NHI Registry for Beneficiaries 2005 and are representative of the complete NHIRD study population. Quality assurance measures have been operationalized and implemented to assure the completeness and accuracy of the NIHRD claims dataset. The Longitudinal Health Insurance Database 2005 consists of de-identified secondary data released to the public for research purposes and was therefore exempted from full review by the Tsaotun Psychiatric Center’s Institutional Review Board. The study adhered to the Declaration of Helsinki and all laws in Taiwan.

### Study samples

We assembled a population-based matched case–control study comprised of individuals who have been participants of the NHI program since January 1, 1997. The study outcome was incidence of glaucoma diagnosis. Cases were identified using the International Classification of Disease, Ninth revision (ICD-9) codes 365.0, 365.1, 365.2, 365.7 and 365.8. These codes include diagnoses of glaucoma suspect, open-angle glaucoma, primary angle-closure glaucoma, glaucoma state and other specified forms of glaucoma. We excluded cases with diagnosis of corticosteroid-induced glaucoma (ICD-9 code 365.3), glaucoma associated with congenital anomalies dystrophies and systemic syndromes (ICD-9 code 365.4), glaucoma associated with disorders of the lens (ICD-9 code 365.5), or glaucoma associated with other ocular disorders (ICD-9 code 365.6). All medical claims made under the foregoing diagnostic codes between 1997 and 2009 were collected from the LHID2005 for further analysis, including inpatient and outpatient services, a definition consistent with other research using this database [22]. For assessing the association between SSRIs exposure and glaucoma, up to five controls were randomly sampled from the remaining samples and matched for each case on demographic variables (e.g. sex, age) and index date.

### Cases of glaucoma and controls

**Fig 1:**
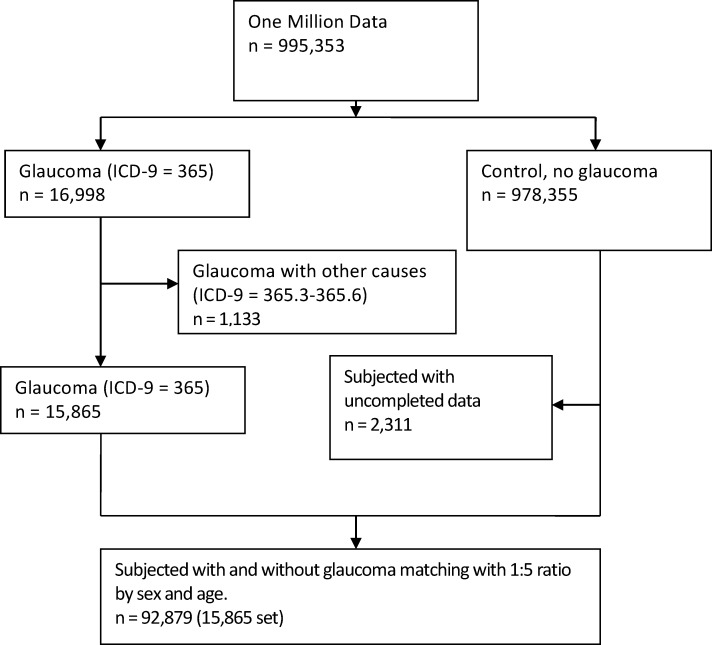
Flow of the study subjects enrollment.

The primary study outcome herein was the incidence of glaucoma. The diagnosis of glaucoma was defined as two or more outpatient diagnoses or one inpatient diagnosis of glaucoma, based on the ICD9-CM codes. For cases, the date of diagnosis of glaucoma was defined as the index date. For controls, the sampled date was used as the index date. For each glaucoma case, we used an incidence density sampling method [23] and randomly selected five controls without a diagnosis of glaucoma. Controls were individually matched to cases by age (i.e. year of birth) and sex. To be included in the nested case-control analysis, an individual must have been free of any diagnosis of glaucoma before the index date (Fig 1).

### SSRIs exposure

We identified SSRIs (N06AB) according to the Anatomical Therapeutic Chemical (ATC) classification system (e.g. citalopram, escitalopram, fluoxetine, fluvoxamine, paroxetine, and sertraline) [24].

The proximity of SSRIs prescription to the diagnosis of glaucoma was assessed using the most recent prescription date before the index date. Exposure to SSRIs before index date was classified dichotomously. In order to explore the dose-response relationship between glaucoma and SSRIs exposure, we calculated the overall duration of use by summing up the length of each prescription. There were three categories of SSRIs cumulative duration before the index date: 1–90, 91–365, and >365 days of SSRIs prescription before the index date. Both dose and duration of SSRIs exposure were assessed with regards to putative association with glaucoma. The dosage of SSRIs was assessed using the defined daily dose (DDD), “the average maintenance dose per day for a drug used for its main indications in adults” [25]. We classified the average DDD into <1 DDD or ≥1 DDD per day.

### Potential confounding factors

We adjusted for potential confounding variables, including age, gender, income, urbanization, hypertension, diabetes and hypercholesterolemia, prescriptions with antipsychotics, anticholinergics, topiramate, steroids, hormone replacement treatment, and other antidepressants: tricyclic antidepressants, serotonin-norepinephrine reuptake inhibitors as possible confounding factors.

### Statistical analysis

Descriptive statistics of glaucoma cases and controls were presented in terms of their demographic characteristics, comorbid disorders, medical procedure and other medication use. We compared the distribution of demographic factors and the proportions of comorbidities between the glaucoma (case) group and non-glaucoma (control) group with unpaired t test for continuous variables and χ^2^ test for categorical variables.

Conditional logistic regression was used to estimate the association between SSRIs treatment and the risk of glaucoma. To understand variable aspects that SSRIs may affect the incidence of glaucoma, we examined the association between risk of glaucoma and average doses and the exposed time-point of SSRIs. In average doses of SSRIs, low (less than 1 DDD) and high (more than 1 DDD) were analyzed. The crude odds ratio (OR) and the adjusted OR were calculated in three SSRIs cumulative duration (1–90, 91–365, and >365 days before glaucoma) of SSRI prescriptions.

Two adjusted models were used. The adjusted model 1 was adjusting for confounding factors including income, urbanization, hypertension, diabetes and hypercholesterolemia. The adjusted model 2 was adjusting for the prescription of non-SSRIs medications including steroid, antihistamine, antipsychotics, anticholinergic drugs and beta-blockers additional to the factors in the model 1. Furthermore, using separate statistical models, we investigated the differential risk for glaucoma with SSRIs use stratified by SSRIs exposure, cumulative duration and cumulative dosage.

We performed subgroup analyses to investigate the potential moderational effect of demographic and clinical characteristics on the risk of developing glaucoma after the exposure of SSRIs. The analysis was stratified by age (i.e. ≤65, >65 years), gender, income, urbanizations and the presence of medical comorbidities (i.e. diabetes, hypertension and hypercholesterolemia). As cases and controls were not matched by clinical characteristics, unconditional logistic regression models with adjustment were performed to evaluate the influence of clinical variation among the study subjects.

The statistical significance of findings was assessed using a 95% confidence interval (CI) or a p value less than 0.05. All analyses were performed using SAS version 9.4 (SAS Institute, Cary, NC).

## Results

### Characteristics of the subjects

15,865 cases with incident glaucoma diagnosis and 77,014 matched controls were included from the database. Cohort characteristics are listed and compared in Table 1. Among the glaucoma cases, 11,573 (72.9%) were under 65 years old. The majority of cases were female (53.3%); residents in high (42.8%) or very high urbanization areas (36.2%, *p*<0.0001); and of lower income strata (27.9%, *p*<0.0001). In comparison, only 28.5% of controls resided in very high urbanization areas. Furthermore, 3360 (21.2%) of glaucoma cases had a diagnosis of type 2 diabetes mellitus, 4874 (30.7%) had hypertension and 2987 (18.8%) had hypercholesterolemia (*p*<0.0001) as a coded diagnosis.

**Table 1:** Characteristics of subjects.

Variable	Glaucoma(n = 15865)	Control(n = 77014)	p-value
	No. (%)	No. (%)	
**Age (years)**			0.39
< = 65	11573 (72.9)	56434 (73.3)	
> 65	4292 (27.1)	20580 (26.7)	
**Sex**			
Female	8456 (53.3)	41306 (53.6)	0.44
**Urbanization**			<0.0001
Low	937 (5.9)	6,560 (8.5)	
Moderate	2381 (15.0)	13385 (17.4)	
High	6791 (42.8)	35109 (45.6)	
Very high	5756 (36.2)	21960 (28.5)	
**Income (NTD)**			<0.0001
0	4423 (27.9)	20526 (26.7)	
1–15840	2559 (16.1)	11589 (15.1)	
15,841–25,000	5895 (37.2)	32300 (41.9)	
>25,000	2988 (18.8)	12599 (16.4)	
**Medical conditions**			
Diabetes	3360 (21.2)	9450 (12.3)	<0.0001
Hypertension	4874 (30.7)	18132 (23.5)	<0.0001
Hypercholesterolemia	2987 (18.8)	9216 (12.0)	<0.0001

### Glaucoma risk by exposure to SSRIs

In comparison to controls, glaucoma cases were significantly more likely to have been exposed to SSRIs (OR = 1.39; 95% CI = 1.29–1.50) (Table 2). The adjusted odds ratio (aOR) remained significant after adjusting for confounding factors including income, urbanization, hypertension, diabetes and hypercholesterolemia (aOR = 1.28; 95% CI = 1.19–1.38). In addition, the elevated risk for glaucoma associated with SSRIs exposure remained significant after adjusting for the prescription of non-SSRIs medications that may be associated with greater risk for glaucoma (e.g. steroid, antihistamine, antipsychotics, anticholinergic drugs and beta-blockers) (aOR = 1.09; 95% CI = 1.00–1.18). The average dose of SSRIs (i.e. ≥1DDD) was significantly associated with an elevated risk for glaucoma (aOR = 1.13; 95% CI = 1.03–1.24). SSRI cumulative duration of >365 days was also associated with elevated risk for developing glaucoma (aOR = 1.25; 95% CI = 1.04–1.50). The combination of >365 days and high (≥1DDD) average dose of SSRIs exposure was additionally associated with greater risk for developing glaucoma (aOR = 1.36; 95% CI = 1.08–1.71) (Table 3).

**Table 2:** Glaucoma risks by exposure status of antidepressant use.

Exposure state	Cases (%)	Controls (%)	Crude OR (95% CI)	Adjusted OR (95% CI)	
	N = 15,865	N = 77,014		Model 1	Model 2
None	14925 (94.1)	73716 (95.7)	1.00	1.00	1.00
Ever	940 (5.9)	3298 (4.3)	1.39 (1.29–1.50)	1.28 (1.19–1.38)	1.09 (1.00–1.18)
**Average dose (DDD/day)**					
<1	307 (1.9)	1129 (1.5)	1.32 (1.16–1.50)	1.19 (1.04–1.35)	1.01 (0.88–1.15)
≥1	633 (4.0)	2166 (2.8)	1.43 (1.31–1.57)	1.34 (1.22–1.46)	1.13 (1.03–1.24)
**SSRI cumulative duration (day)**					
1–90	545 (3.4)	2025 (2.6)	1.32 (1.20–1.45)	1.26 (1.14–1.39)	1.07 (0.97–1.19)
91–365	214 (1.4)	778 (1.0)	1.62 (1.15–1.56)	1.20 (1.02–1.40)	1.02 (0.87–1.19)
> 365	181 (1.1)	495 (0.6)	1.77 (1.49–2.10)	1.51 (1.26–1.80)	1.25 (1.04–1.50)

OR, odd ratio; CI, confidence interval.

^a^Model 1: adjusting with income, urbanization, hypertension, diabetes and hypercholesterolemia.

^b^Model 2: adjusting with income, urbanization, hypertension, diabetes, hypercholesterolemia and medications (steroid, antihistamine, antipsychotics, anticholinergic drugs and beta-blocker).

**Table 3:** Glaucoma risk by exposure status of antidepressant use in the low (DDD < 1) and high (DDD ≥ 1) average doses.

SSRI cumulative duration (day)	Cases (%)	Controls (%)	Crude OR (95%CI)	Adjusted OR (95% CI)	
	N = 15865	N = 77014		Model 1^a^	Model 2^b^
**Low (DDD < 1) average dose**					
0			1.00	1.00	1.00
1–90	157 (1.0)	583 (0.8)	1.31 (1.10–1.56)	1.23 (1.03–1.48)	1.05 (0.87–1.26)
91–365	82 (0.5)	335 (0.4)	1.20 (0.94–1.53)	1.04 (0.82–1.34)	0.89 (0.69–1.14)
> 365	68 (0.4)	211 (0.3)	1.55 (1.18–2.04)	1.29 (0.98–1.71)	1.10 (0.83–1.46)
**High (DDD ≥ 1) average dose**					
0			1.00	1.00	1.00
1–90	388 (2.5)	1439 (1.9)	1.32 (1.18–1.48)	1.27 (1.13–1.43)	1.09 (0.97–1.22)
91–365	132 (0.8)	443 (0.6)	1.45 (1.20–1.77)	1.31 (1.08–1.60)	1.12 (0.91–1.36)
> 365	113 (0.7)	284 (0.4)	1.94 (1.56–2.42)	1.67 (1.33–2.09)	1.36 (1.08–1.71)

OR, odd ratio; CI, confidence interval.

^a^Model 1: adjusting with income, urbanization, hypertension, diabetes and hypercholesterolemia.

^b^Model 2: adjusting with income, urbanization, hypertension, diabetes, hypercholesterolemia and medications (steroid, antihistamine, antipsychotics, anticholinergic drugs and beta-blocker).

### Subgroup analysis of the glaucoma risk with SSRIs treatment

We performed subgroup analyses stratifying by age (i.e.≤65, >65 years), gender and medical comorbidities (Table 4). Regardless of age, both males and females were at significantly higher glaucoma risk with SSRIs exposure. The subgroup analysis indicated that the risk of SSRIs with glaucoma was noted in individuals younger than 65 years of age (aOR = 1.37; 95% CI = 1.25–1.50), without diabetes (aOR = 1.39; 95% CI = 1.27–1.52), without hypertension (aOR = 1.46; 95% CI = 1.31–1.63), without hypercholesterolemia (aOR = 1.35; 95% CI = 1.23–1.48), and with high urbanization across income categories.

**Table 4:** Subgroup analysis of glaucoma risk and SSRI use by patient characteristics.

Variable	Case		Control		Crude OR (95% CI)	Adjusted OR (95% CI)
	User	Non-user	User	Non-user		
Age*						
≤ 65 (n = 68007)	691	10882	2,289	54145	1.50 (1.37–1.64)	1.37 (1.25–1.50)
>65 (n = 24872)	249	4,043	1009	19571	1.16 (1.01–1.34)	1.09 (0.94–1.26)
Sex*						
Female (n = 49762)	583	7873	2,118	39188	1.36 (1.23–1.49)	1.26 (1.14–1.39)
Male (n = 43117)	357	7052	1,180	34528	1.45 (1.29–1.64)	1.32 (1.16–1.49)
Urbanization**						
Low	57	880	323	6237	1.43 (0.72–2.84)	1.32 (0.66–2.67)
Moderate	146	2235	573	12812	1.43 (1.05–1.95)	1.38 (1.01–1.90)
High	383	6180	1,440	32662	1.42 (1.23–1.64)	1.31 (1.14–1.52)
Very high	333	5423	911	21049	1.59 (1.34–1.89)	1.49 (1.25–1.77)
Income (NTD)**						
0	222	4201	756	19770	1.41 (1.14–1.73)	1.31 (1.06–1.62)
1–15840	169	2390	574	11015	1.43 (1.09–1.89)	1.38 (1.04–1.82)
15841–25000	386	5509	1,469	30831	1.39 (1.21–1.59)	1.26 (1.10–1.45)
>25,000	163	2825	499	12100	1.50 (1.16–1.93)	1.37 (1.05–1.78)
Diabetes***						
Yes (n = 12810)	269	3091	739	8711	1.09 (0.87–1.34)	1.08 (0.87–1.35)
No (n = 80069)	671	11834	2559	65005	1.46 (1.33–1.60)	1.39 (1.27–1.52)
Hypertension***						
Yes (n = 23006)	440	4434	1,458	16674	1.09 (0.95–1.25)	1.06 (0.93–1.22)
No (n = 69873)	500	10491	1,840	57042	1.53 (1.37–1.70)	1.46 (1.31–1.63)
Hypercholesterolemia***						
Yes (n = 12203)	286	2701	825	8391	1.13 (0.93–1.38)	1.13 (0.93–1.39)
No (n = 80676)	654	12224	2,473	65325	1.42 (1.29–1.56)	1.35 (1.23–1.48)
**Depressive disorder**						
Yes (n = 3428)	475	312	1485	1156	1.07 (0.71–1.63)	1.13 (0.72–1.75)
No (n = 89451)	465	14613	1813	72560	1.25 (1.13–1.39)	1.18 (1.06–1.31)

OR, odd ratio; CI, confidence interval.

*Adjusting with income, urbanization, hypertension, diabetes and hypercholesterolemia.

**Adjusting with sex, age, hypertension, diabetes and hypercholesterolemia.

***Adjusting with income, urbanization.

## Discussion

In this population-based, nested case-control study, we found that individulas prescribed SSRIs had only weak association with the risk of glaucoma (aOR = 1.09; 95% CI = 1.00,1.18). Longer duration of SSRIs treatment and higher average dose of SSRIs were associated with greater risk for glaucoma with an additive increased risk when both of these foregoing variables were included in the model. Furthermore, the effect of SSRIs on glaucoma was limited to individuals younger than 65 years old, without diabetes, hypertension or hypercholesterolemia.

The foregoing observation (i.e. risk between SSRI exposure and increased glaucoma hazard) comports with results from several published studies [11,29]. For example, several studies have reported that fluoxetine is associated with significant elevation in intraocular pressure (IOP) in both animals and humans [3,26]. Case reports also suggest that serotonin agonists and antagonists directly alter IOP [27,28]. SSRIs have relatively weak anticholinergic and adrenergic effects but can directly influence the iris or the ciliary body muscle perhaps mechanistically providing an understanding of the association.

Serotonergic neurons are found in the raphe nuclei and project to various effector neurons, including motor neurons in the mammalian eye [29]. Serotonin and its receptors have been identified in different ocular structures and are involved in various functional role of ocular tissues, including the cornea, iris, ciliary muscle, iris sphincter muscle, lens and retina. For example, 5-HT_1A_ receptors in the iris and ciliary body were found to reduce the production of aqueous humor, reducing intraocular pressure. It could be conjectured that subchronic/chronic dosing of SSRIs could possibly increase intraocular pressure via desensitization of 5-HT_1A_ receptors. Similarly, 5-HT_7_ receptors can augment the production of aqueous humor and relax the sphincter of the pupil and consequently induce mydriasis. The 5-HT_2_ subtype receptor has a major role in the regulation of fluid transport or corneal homeostasis. Extant literature has reported on the presence of serotonin and its metabolites in the aqueous humor, together with 5-hydroxytryptamine receptors in the iris–ciliary body complex [3]. It is hypothesized that serotoninergic enhancement may induce mydriasis and narrow angle conformation. The foregoing would be expected to block the aqueous circulation, elevate intraocular pressure, and subsequently induce secondary glaucoma [30].

We also found that the overall exposure to SSRIs, as defined by cumulative duration and/or average daily dose, was associated with higher incidence of glaucoma. With regards to temporality, previous case studies have reported that the onset of glaucoma after commencing SSRIs treatment ranges from several days to weeks and was generally less than 6 months [13,30,31]. Our results show the risk of developing glaucoma within 1–90 days, 91–365 days and >365 days of SSRIs prescription was significant after adjusting for income, urbanization, hypertension, diabetes and hypercholesterolemia. However, after adjusting for prescription of concomitant medications that might influence glaucoma risk, only long-term use of SSRIs prescription (>365 days) was significant. Chen *et al* found long-term use of SSRIs in depression patients does not influence the risk of glaucoma [15]. It should be noted, however, that the study population evaluated by Chen et al. was delimited to those with Major Depressive Disorder (MDD). As SSRIs are commonly prescribed off-label for a wide range of conditions, the generalizability of the foregoing findings may be limited. For example, the proportion of antidepressants prescribed for individuals without any psychiatric diagnoses were noted to have increased from 59.5 percent to 72.7 percent in the United States [16]. The interpretation of available studies is affected by the fact that SSRIs prescribed across disparate medical and psychiatric populations, which may influence the overall risk of developing glaucoma with SSRI exposure.

We noted that treatment with SSRIs increases the risk of developing glaucoma at higher (i.e., ≥1 DDD) doses. Our finding is consistent with the study by Chen et al. that reported a significant elevation in glaucoma risk among participants receiving a mean daily dose of SSRIs exceeding 20 mg [14]. The foregoing finding suggests that precaution must be taken to prevent progression to glaucoma with SSRI exposure. However, to our knowledge, no previous research has reported on the dose-dependent effects of long-term SSRIs exposure and glaucoma risk.

General medical condition is considered to be an important factor contributing to the risk of developing glaucoma. Herein, we found an increased risk on glaucoma with SSRI prescription only in the subgroups without diabetes, hypertension, and hypercholesterolemia. We conjecture that glaucoma onset in individuals with comorbid diabetes, hypertension, and hypercholesterolemia are not associated with SSRIs exposure but more related to their underlining health condition.

Available evidence indicates that SSRIs-induced glaucoma is most common among individuals between the ages of 32 and 70 years [13]. Older age is an established risk factor for glaucoma [32]. Herein, we report that the effect of SSRIs on glaucoma was found on individuals aged less than 65 is elevated with exposure to SSRIs; an effect that persisted after adjusting for potentially confounding concomitant medications. Replication of the foregoing finding is however needed before definitive conclusions can be made. Multiple contacts with physicians might increase the chance of patients being prescribed SSRIs with an associated increased risk for an additional diagnosis, of glaucoma. Hence, we conducted the sensitivity analysis by classifying participants into tertiles according to the number of outpatient visits before the index date. There were still higher risk for glaucoma in patients with the SSRI ever use (aOR = 1.09; 95% CI = 1.00–1.17), high average dose of SSRI (aOR = 1.13; 95% CI = 1.03–1.24) and longer SSRI cumulative duration (aOR = 1.27; 95% CI = 1.06–1.51).

In summary, our study indicates that individuals receiving SSRI therapy may be at elevated risk for glaucoma particularly at higher doses and with long-term use. Further studies are necessary to determine if SSRI exposure causal and what are the mechanistic pathways. Patients should be educated about the possible risk of developing glaucoma with SSRI treatment, as well as about possible symptoms of glaucoma to assure early detection and intervention.

### Limitations and strengths

There are several limitations to this study. First, the diagnoses of glaucoma and other comorbid medical conditions were obtained from Taiwan’s National Health Insurance database and diagnoses were proxied by International Classification of Diseases codes, which may be less accurate than formal diagnoses made through standardized examinations and procedures. Second, as our database is comprised of claims data, we did not have access to laboratory or imaging data in individual chart records. Individuals with asymptomatic glaucoma may not have been included in our analysis, which would lead to an underestimation of the effect of SSRIs on the risk of developing glaucoma. Third, we also did not have data on other potentially confounding variables, such as smoking, medication adherence and whether the hypertension, diabetes, and hypercholesterolemia were controlled with the medications prescribed. The result is not able to determine the causality and should be interpreted with caution. Fourth, the study cohort predominantly consisted of Taiwanese individuals; thus these findings may not be easily generalizable to other population groups.

Notwithstanding the aforementioned limitations, the comprehensive nature of the Taiwan’s National Health Insurance database limits selection bias and recall bias. In addition, the large sample size of our analysis is representative of the national population and allows for a sufficiently powered analysis of our primary outcome (i.e. glaucoma incidence).

In conclusion, this population-based case-control study found that SSRIs use was associated with the risk of glaucoma, especially for those with high doses (i.e., ≥1 DDD) and a cumulative duration of >365 days of SSRIs treatment. Further studies will be required to elucidate the risk of glaucoma among other classes of antidepressants and investigate the underlying mechanism.
